# Topology of turbulence within collisionless plasma reconnection

**DOI:** 10.1038/s41598-023-45650-x

**Published:** 2023-10-31

**Authors:** Bogdan Hnat, Sandra Chapman, Nicholas Watkins

**Affiliations:** 1 Physics Department, Centre for Fusion Space and Astrophysics, University of Warwick, Coventry, UK; 2 Department of Mathematics and Statistics, University of Tromsø, Tromsø, Norway; 3 International Space Science Institute, Bern, Switzerland; 4 Grantham Research Institute on Climate Change and the Environment, London School of Economics and Political Science, Houghton Street, London, UK; 5 School of Engineering and Innovation, The Open University, Milton Keynes, UK

**Keywords:** Astrophysical plasmas, Magnetospheric physics

## Abstract

In near-collisionless plasmas, which are ubiquitous in astrophysics, entropy production relies on fully-nonlinear processes such as turbulence and reconnection, which lead to particle acceleration. Mechanisms for turbulent reconnection include multiple magnetic flux ropes interacting to generate thin current sheets which undergo reconnection, leading to mixing and magnetic merging and growth of coherent structures, unstable reconnection current layers that fragment and turbulent reconnection outflows. All of these processes act across, and encompass, multiple reconnection sites. We use Magnetospheric Multi Scale four-point satellite observations to characterize the magnetic field line topology within a single reconnection current layer. We examine magnetopause reconnection where the spacecraft encounter the Electron Diffusion Region (EDR). We find fluctuating magnetic field with topology identical to that found for dynamically evolving vortices in hydrodynamic turbulence. The turbulence is supported by an electron-magnetohydrodynamic (EMHD) flow in which the magnetic field is effectively frozen into the electron fluid. Accelerated electrons are found in the EDR edge where we identify a departure from this turbulent topology, towards two-dimensional sheet-like structures. This is consistent with a scenario in which sub-ion scale turbulence can suppress electron acceleration within the EDR which would otherwise be possible in the electric field at the X-line.

## Introduction

Collisionless magnetic reconnection^1^ and plasma turbulence^2,3^ are fundamental mechanisms that transfer energy across scales and between electromagnetic fields and particles^4,5^. Magnetic field topology is central to both these processes. In two dimensions, reconnection is mediated by structures and interactions that are topologically constrained. In a turbulent flow, multiple reconnection X-lines can co-exist^6^. In such a two-dimensional flow, magnetic flux islands interact to generate thin current sheets^7^ which undergo reconnection, leading to magnetic merging and growth of coherent structures^8–10^. A given reconnection current layer is also unstable and tends to fragment into a population of secondary magnetic islands and current filaments^11–13^, even when embedded in a laminar flow. These processes are seen both in direct numerical simulations^11,14^ and in *in-situ* observations^15,16^. Three-dimensional reconnection differs significantly from its two-dimensional counterpart due to turbulence^6,17,18^ and asymmetries of the reconnecting current sheet^19,20^. Fully kinetic three dimensional simulations of reconnection reveal a highly tangled flux rope structure^21^ and outflow regions that can become turbulent^22^. All of these processes are either turbulence in an accelerated outflow, or are the generation of, or interaction between, multiple X-lines or magnetic null points.

Here, we classify the magnetic field topology observed as the four MMS spacecraft fly through a well resolved reconnection site. These observations allow us to quantify the topology of turbulence *within* a single reconnection layer. *In-situ* field and plasma observations from a tetrahedron of four spacecraft can be combined to directly classify the magnetic field line topology^23–26^ which is fully characterised by the eigenvalues of the magnetic field gradient tensor near a magnetic null^27^. The MMS spacecraft separation defines a spatial ‘yardstick’, which is of order of the ion inertial range $d_i$, for sampling magnetic field topology. Crucially, whilst the magnetic field gradients, and the corresponding magnetic topology, are directly spatially resolved on the scale of the spacecraft separation, how the topology varies spatially is indirectly captured on a much finer spatial scale with the fast sampling in time of the magnetic field simultaneously at all four spacecraft. Within a given plasma region this gives many such samples since the cadence of the observations is fast (magnetic field measurements at 8192 Hz). We will use this high cadence to track how the magnetic field topology varies as the spacecraft move through the reconnection region. We obtain the time evolution of the magnetic field gradient tensor (MFGT) invariants, including, for the first time, those related to the magnetic field deformation (strain rate) tensor. When the spacecraft encounter the Electron Diffusion Region (EDR), we identify a particular evolution of the topology of the disordered magnetic field, for which the MFGT invariants explore a trajectory that parallels that seen in systems governed by the Euler equations such as experimental realizations of hydrodynamic turbulence^28,29^. In the sub-MHD scales of the MMS reconnection encounter, plasma turbulence is supported by an electron-magnetohydrodynamic (EMHD) flow in which the magnetic field is effectively frozen into the electron fluid^7^.

In this MMS reconnection encounter, accelerated electrons are found at the edge of the EDR, where the magnetic topology becomes sheet-like rather than turbulent. The absence of turbulence would permit direct acceleration of suprathermal electrons at the EDR edge. Heliospheric observations do not unambiguously confirm strong reconnection-driven electron acceleration; whilst it is seen at solar flares^30,31^ and in Earth’s magnetotail^32^, magnetosheath reconnection produces only mildly energised electron jets at a few Alfvén velocities which are often observed near, but not at, the reconnection X-line^33,34^. Our results are consistent with a scenario in which sub-ion scale turbulence can suppress electron acceleration within the EDR which would otherwise be possible in the electric field at the X-line.

**Figure 1:**
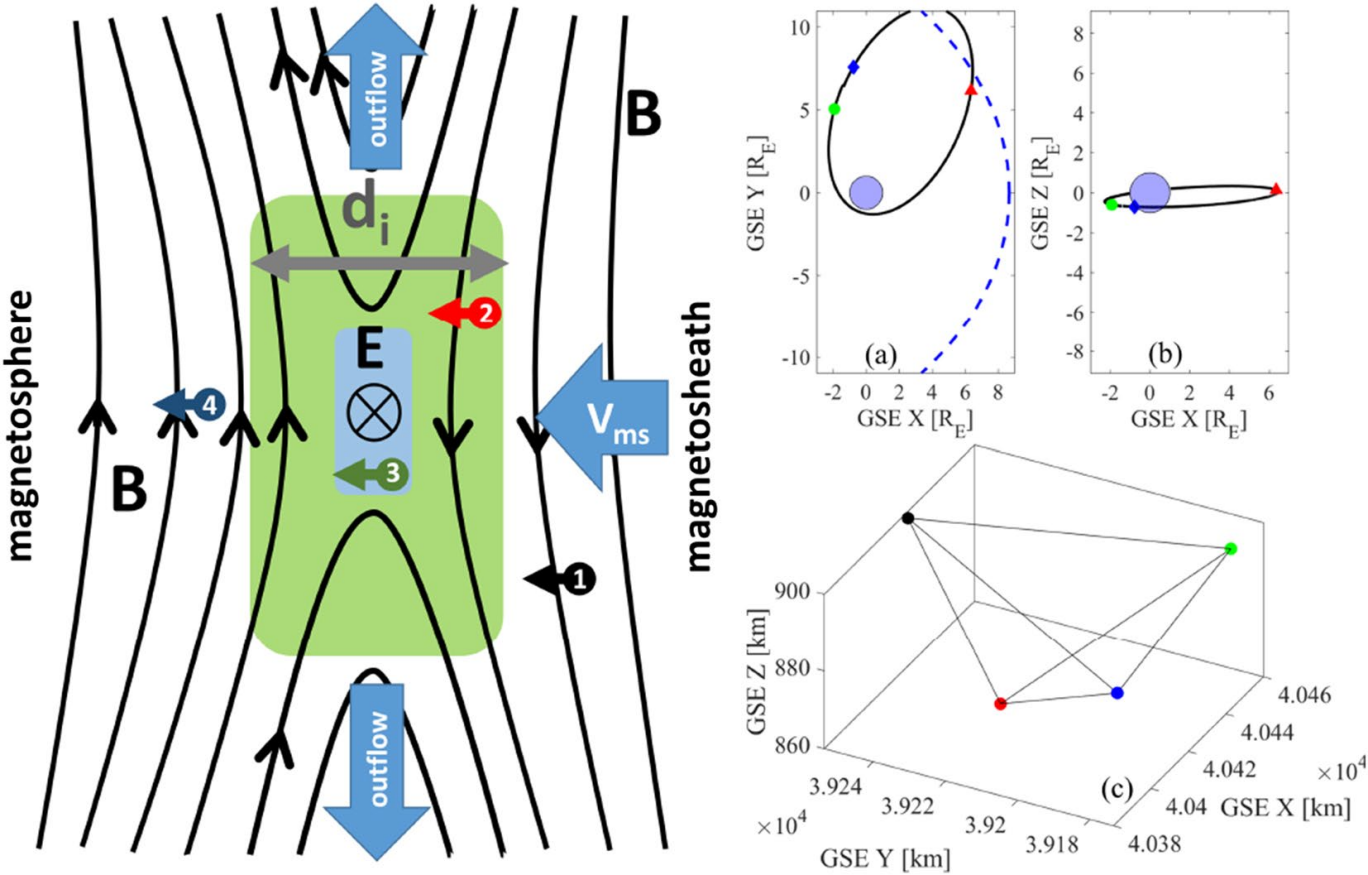
Overview of the observed reconnection region. Left: Schematic of event, showing the magnetic null, the Electron Diffusion Region (EDR) (blue) and surrounding region on the scale of the ion inertial length $d_i$ (green). Numbered circles indicate the locations of MMS spacecraft 1–4 at one time snapshot. Right: the MMS orbit and the MMS spacecraft formation for the date of interest. Panel (**a**) plots the MMS orbit projection onto the GSE x–y plane. The red triangle shows the MMS location at the time of reconnection event. The green circle and blue diamond correspond to the start and the end of the orbit on 19-09-2015, at times 01:00 and 23:00, respectively. The dashed blue curve is the approximate location of the magnetopause at the time of reconnection. Panel (**b**) plots the MMS orbit projection on the GSE x–z plane, symbols are the same as these in panel (**a**). Panel (**c**) shows the spacecraft tetrahedron configuration at the time of reconnection, the MMS spacecraft 1–4 positions are shown in black, red, green and blue, respectively.

## Results

Figure 1 is an overview of the MMS fly-through of a magnetosheath reconnection site^35–38^. The left panel sketches a simplified 2D schematic showing regions of plasma inflow and outflow centered on a magnetic field null or X-line. The overall physical picture of this isolated reconnection site is of a magnetohydrodynamic (MHD) flow which sweeps magnetic flux into the inflow region, where MHD breaks down and the flux reconnects at the X-line within the small EDR marked by blue shaded rectangle. The process generates stretched field lines in the outflow region which relax, accelerating a plasma exhaust. Apart from energization in the outflow region, particle acceleration relies on physics on sub-MHD scales within the central current sheet. On scales between the ion and electron inertial lengths, $d_i\!>\! d \!> \!d_e$, marked by the green shaded rectangle, the ions are demagnetized and the magnetic flux is “frozen” into the EMHD flow. Magnetic field observations from the full spacecraft tetrahedron, with spacing $D_{sc}\! \sim \!70$ km or $\sim \! d_i$, then characterize the topology of the magnetic field embedded in this EMHD flow. For this event, the spacecraft formation had high Tetrahedron Quality Factor at 0.95. Coloured circles with numbers indicate the positions of MMS spacecraft 1–4 at one time snapshot. MMS 4 is the closest to the Earth and may have encountered the EDR prior to the other spacecraft, however, not all data signatures are conclusive in supporting this encounter. A previous study found evidence of secondary flux ropes produced by the Kelvin–Helmholtz instability during the ion diffusion region crossing by MMS 4^38^, which could imply the existence of multiple EDRs within the interval considered here. While results presented below are consistent with the presence of such flux ropes, the accuracy of the magnetic field measurements does not allow us to draw strong conclusions as to the presence of the multiple X-lines at these times. Among MMS spacecraft 1, 2 and 3, MMS 3 encounters the reconnection layer first. The right-hand panels of Fig. 1 show the MMS 3 orbit and the spacecraft formation on the day of interest. The red triangle in panels (a) and (b) shows the MMS3 location at the time of the reconnection event. The spacecraft were located towards the dusk flank of the magnetopause, at the distance of approximately $8.8R_E$ from the Earth.

**Figure 2:**
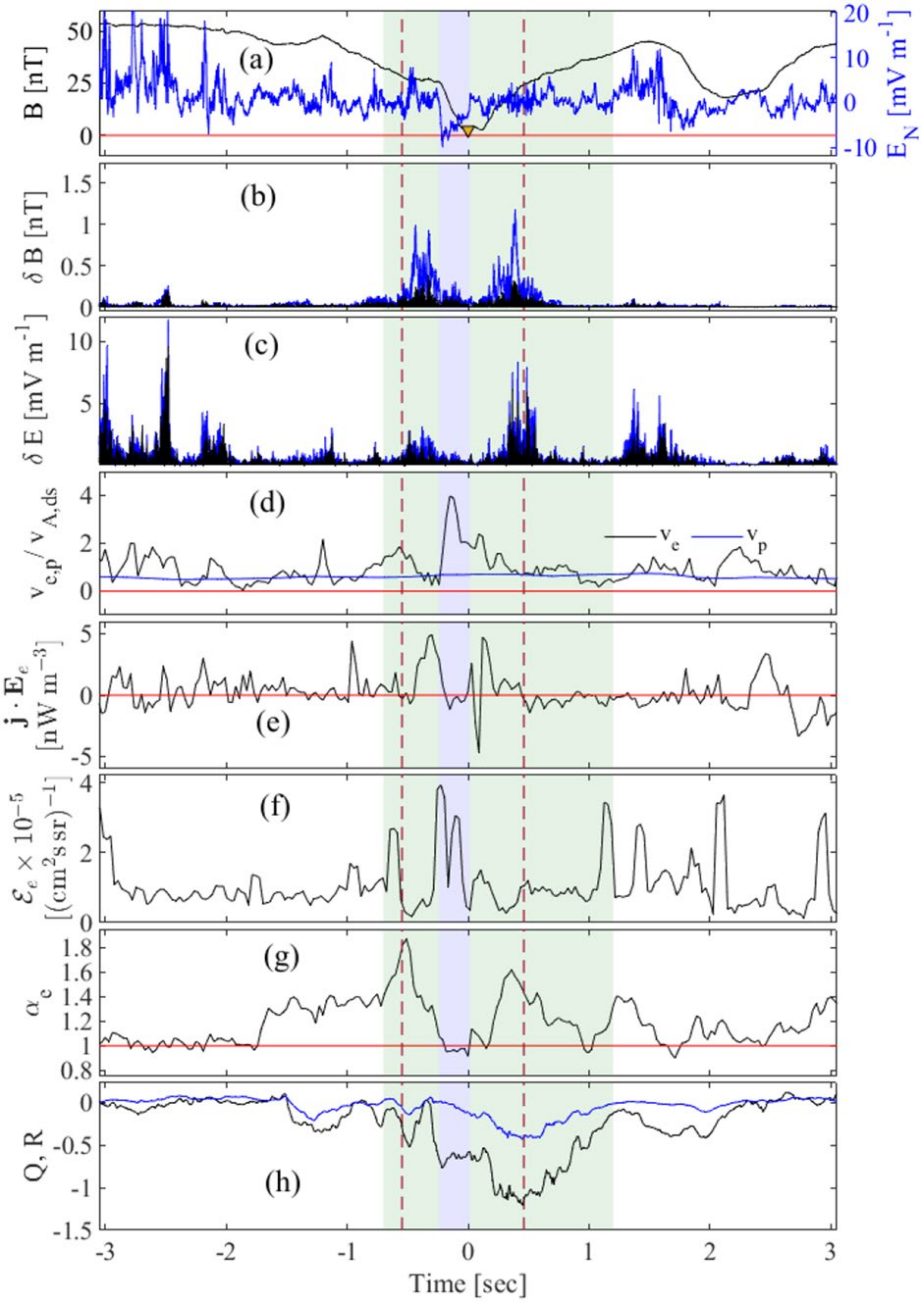
MMS 3 observations of the reconnection region. Time series of *in situ* observations of the reconnection region transit seen by MMS 3, which leads MMS 1 and 2 in the encounter. The EDR is indicated with blue shading on all panels. Green shading indicates the time interval in which at least one spacecraft samples the EDR. We will focus on the magnetic topology within these regions in Figs. 3 and 4. Reading from top to bottom, the Figure plots: in panel (**a**) magnetic field magnitude (black), the magnetic null is at 19-Sep-2015 at 07:43:30.488, used throughout to define $t=0$ indicated by the yellow triangle. Electric field component $E_N$ (blue) in the event LMN coordinates defined in^35^ (blue) shows a negative excursion which locates the EDR^35^. Panel (**b**) plots band pass filtered magnetic field components (blue) and magnetic field magnitude (black) within frequency range 64–256 Hz; the dashed red vertical lines mark the outer extent of large magnetic field fluctuations. Panel (**c**) plots the same quantity as panel (**b**) calculated for the electric field fluctuations. Panel (**d**) plots electron flow speed (blue) which peaks within the EDR, and ion flow speed (black) which is approximately constant, both are normalised to the downstream Alfvén speed. Panel (**e**) plots electron heating $\varvec{j} \cdot \varvec{E}_e$ which peaks at the edges of the EDR. Panel (**f**) plots suprathermal electron flux, $\mathcal {E}_e$, defined as the differential electron flux integrated over all angles and over energy range 800–$1\times 10^4$ eV, which peaks within the EDR. Panel (**g**) plots bulk electron temperature anisotropy, $\alpha _e$, which peaks coincide with enhanced magnetic fluctuations. Panel (**h**) shows the main invariants, *Q* and *R*, of the MFGT.

The time series from a single spacecraft is at sufficiently high cadence to capture features at the scale of the electron inertial length. The time series from spacecraft MMS 3 is shown in Fig. 2. This MMS 3 encounter with the EDR on the 19 of September 2015 at around 07:43:30 has been previously documented^35–38^, see Methods for details. The relevant features of this encounter with the reconnection region are as follows. The magnetic field null is encountered by MMS 3 at 07:43:30.488, and this time is used throughout to define $t=0$. The MMS encounter with the reconnection site, that is, the time interval in which at least one MMS spacecraft is within the EDR region, is indicated by green shading across all panels. Blue shading across all panels indicate times when MMS 3 is within the EDR.

In Fig. 2, panel (a) plots the magnetic field magnitude (black), and the electric field component $E_N$ (blue) in the event LMN coordinates defined as described in^35^, a more general description can be found in^18^. In the vicinity of the reconnection region, there is a coherent negative excursion of the electric field which defines the EDR^35^, this is offset from the magnetic field null (yellow triangle). There are strong fluctuations in both the electric and magnetic field in the vicinity of the EDR. The fluctuations in the magnetic field components (blue) and magnitude (black) of the combined FGM/SCM data, captured by a band pass filter with frequency range 64–256 Hz, are plotted in panel (b). These fluctuations are a combination of turbulent electron motion as well as whistler waves^37^, as evident from the power spectral density of magnetic field components (see figure 4 of the SI document). The magnetic components fluctuate strongly in the vicinity of the EDR (time interval indicated by red dashed lines) and there is a suppression of magnetic field magnitude variation within the EDR. Panel (c) plots the electric field fluctuations obtained with the same method as those in panel (b). The electric field fluctuates strongly in the vicinity of the EDR and these fluctuations are suppressed within the EDR. The bulk electron flow speed is enhanced in the EDR whereas the bulk proton flow remains approximately constant and sub-Alfvénic throughout, as shown in panel (d). There is evidence of electron heating at the edge of the EDR seen in $\textbf{j} \cdot \textbf{E}_e$ calculated in the electron frame of reference (see Methods), shown in panel (e). Suprathermal electrons coincide with the excursion of the electric field within the EDR, shown in panel (e) which plots differential electron flux integrated over all angles and over the energy range 800–$1\times 10^4$ eV. Finally, the bulk electron anisotropy is enhanced where the magnetic field most strongly fluctuates, just outside the EDR. Within the EDR, the bulk electrons are essentially isotropic. The observed change in the electron temperature anisotropy is rather modest, with a maximum at about $60\%$ of the mean. Finally, Panel (h) plots time series of the MFGT invariants *Q* and *R* which we discuss next.

This set of time series from MMS 3 provides a clear chronology of the distinct physical regions of a well resolved single reconnection layer. However, if we combine magnetic field observations from all four MMS spacecraft, we can classify the magnetic topology sampled as the spacecraft fly through the distinct physical regions, using timings from MMS 3. The magnetic field observed at four MMS spacecraft is used to construct the MFGT $\varvec{M}(t)$. We have applied two different methods, one based on a direct matrix inversion^39, 40^ and another based on nonlinear least squares optimisation^41,42^ (the lsqnonlin function in Matlab). We have found that the least squares method gives an MFGT which satisfies $\nabla \cdot \textbf{B} = 0$ to much higher accuracy. All results presented below are obtained using the least squares optimisation. We give a more detailed description of these two approaches in the Magnetic Field Gradient Tensor section. The topological invariants, *R* and *Q*, of this tensor characterise the magnetic field line topology^23,25,26^.

**Figure 3:**
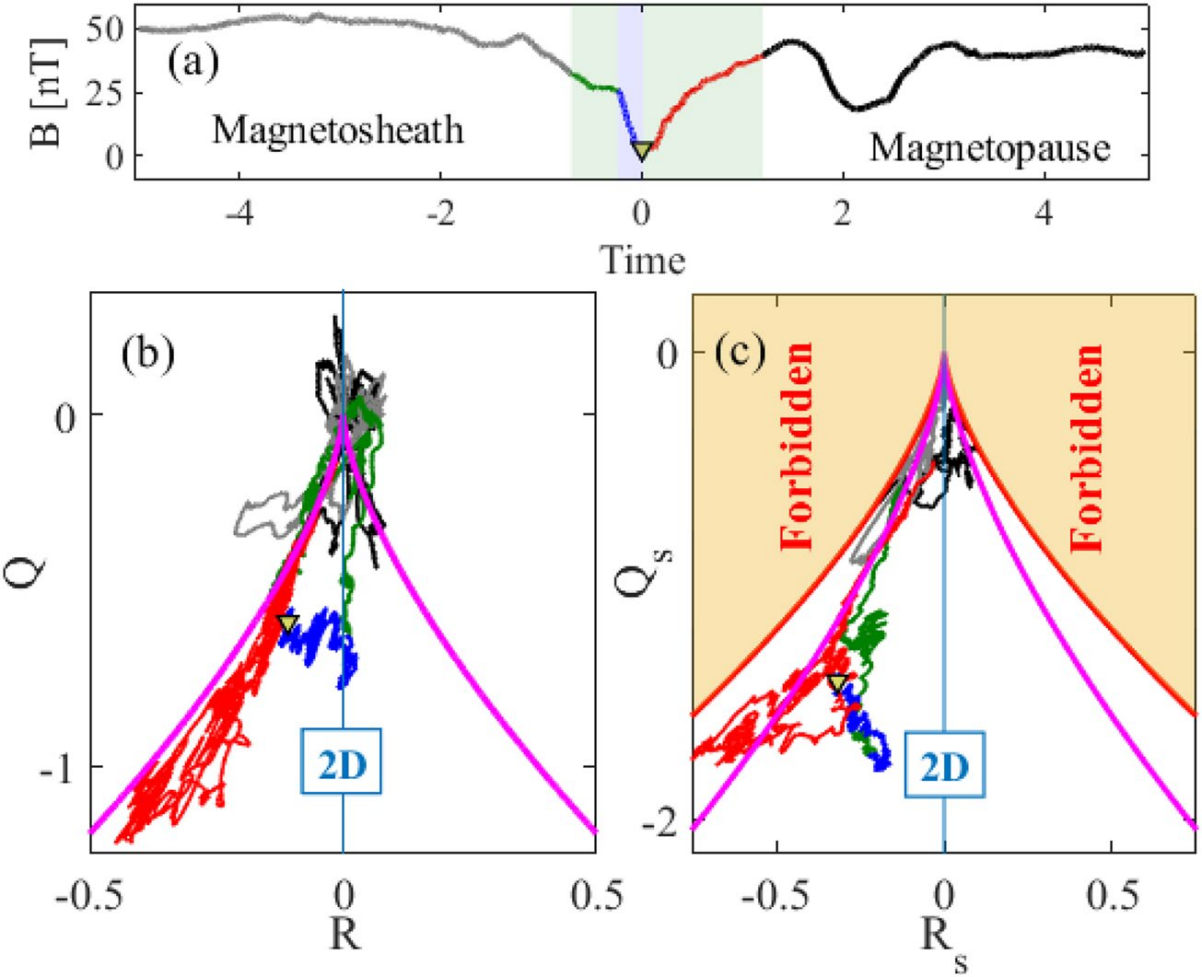
Magnetic field topology within the reconnection region. Panel (**a**) reproduces the information of Fig. 2a and is used to colour-code specific time intervals: green shading (as in Fig. 2) indicates the time interval in which at least one spacecraft samples the EDR, split into sub-intervals indicated by the dark green, blue and red traces, which are the leading edge of the EDR, the EDR, and the trailing edge of the EDR respectively. Regions before, and after the EDR is encountered are indicated by grey and black time traces respectively. The same colours are used in the lower panels to indicate the time intervals within which the topological invariants are obtained. The lower panels plot the topological invariants for all field deformations *R*, *Q* (panel **b**) and the curl-free field deformations $R_s,Q_s$ (panel **c**), respectively. In panel (**b**) elliptic $\Delta >0$ (flux ropes) and hyperbolic $\Delta <0$ are seperated by the magenta line $\Delta =0$. In panel (**c**), $\Delta _s=0$ is the boundary of possible curl-free deformations of magnetic field lines. The magenta line in panel (**c**) corresponds to triaxial deformations with eigenvalue ratios − 3:− 1:4 ($R_s<0$) and 3:1:− 4 ($R_s>0$), as found in the strain tensor of a three dimensional hydrodynamic flow^28,29^.

The symmetric part of $\varvec{M}$, $\varvec{S}$, with invariants $R_s$ and $Q_s$, relates to a rate of strain of magnetic field, that is, all curl-free deformations of the magnetic field lines which are not related to a current. All invariants are normalised by the power in the current $(\mu _0 j)^2$ (see Methods). As the four spacecraft travel through the reconnection region, they provide multiple samples of the magnetic field topological invariants and these are plotted in Fig. 3. Figure 3 is structured as follows: Panel (a) plots the magnetic field magnitude measured by MMS 3 over a slightly extended time interval compared with Fig. 2a, with the field null (yellow triangle) at $t=0$. Green shading again indicates the time interval in which at least one MMS spacecraft samples the EDR. The EDR region is indicated by blue shading. The magnetic field magnitude time trace is color coded to indicate sub-intervals of the encounter: dark green, blue and red traces respectively indicate the leading edge of the EDR, the EDR, and the trailing edge of the EDR, respectively. The regions before and after the EDR is encountered are indicated by grey and black time traces, respectively. These colors are used throughout Fig. 3 to refer to MMS’s encounter with different regions of the reconnection site.

This high resolution time series of topological invariants can then be plotted in the invariant space, for the field gradient tensor $\varvec{M}$ in *R*, *Q* space (panel (b)) and for the deformation tensor $\varvec{S}$, in $Q_s(t),R_s(t)$ (panel (c)). The overall magnetic field topology in *R*, *Q* space is discriminated by the contour $\Delta \!=\!(27/4)R^2+Q^3$ (see Methods). Elliptic magnetic field lines, that is, flux ropes, satisfy $\Delta >0$, whereas for $\Delta <0$ the field lines are hyperbolic, forming a multi-pole separatrix structures consistent with 3D reconnection (3D X-line). In terms of the eigenvalues $\lambda ^s_1$, $\lambda ^s_2$, $\lambda ^s_3$ of the deformation tensor ***S***, $R_s \!= \!-\lambda ^s_1 \lambda ^s_2 \lambda ^s_3$ and $Q_s \!= \! \lambda ^s_1 \lambda ^s_2 + \lambda ^s_2 \lambda ^s_3 + \lambda ^s_1 \lambda ^s_3$. In $R_s$, $Q_s$ space, the contour $\Delta _s=0$ (where $\Delta _s \!=\!(27/4)R_s^2+Q_s^3$) is the boundary of curl-free deformations of the magnetic field, corresponding to eigenvalue ratios 2:$-1$:$-1$ ($R_s<0$) and 1:1:$-2$ ($R_s>0$). The topology tends to two-dimensional, stretched field lines in the vicinity of $R=0$ and $R_s=0$. The sign of *R* and $R_s$ is determined by the overall magnetic field polarity through the reversal.

We now use Fig. 3 to track how the magnetic field topology evolves as the MMS spacecraft tetrahedron transits the reconnection site. Panel (b) tracks the overall topology and at times well outside the EDR and the encounter (grey and black points), the topology of structures in the magnetic field have $\Delta$ just above zero and $R\simeq 0$, that is, they are quasi 2D flux ropes. This is consistent with the previous findings of secondary flux ropes at the time of MMS 4 encounter with the ion diffusion region^38^ (grey points). The brief incursion of black points below the $\Delta =0$ separatrix corresponds to a second minimum of the magnetic field strength at $t\! \approx \! 2$ seconds after the magnetic null is encountered by MMS 3. Within the encounter, at the leading edge of the EDR (green trace), the topology tracks the $\Delta =0$ separatrix and then transitions into hyperbolic field topology region $\Delta <0$. Within the EDR region (blue traces) the topology is consistently X-line type ($\Delta <0$) and is closer to $R\simeq 0$, indicating reduced dimensionality of magnetic field in one of the directions. The field null (yellow triangle) is also well within ($\Delta <0$). The overall topology shown in panel (b) is then just that expected for an encounter with a reconnection region, outside of the encounter, it is flux-rope like, and during the encounter, separatrix-like. The main MFGT invariants are not expected to vary in time at magnetic null point^25^, as can be seen to be the case in the time trace of these invariants plotted in panel (h) of Fig 2.

The invariants $R_s,Q_s$ of curl-free deformations are well known to be informative in the study of *hydrodynamic* turbulence^28,29^. Here, we obtain these for the first time for the *magnetic field*; these are plotted in panel (c). During the MMS encounter with the reconnection site (green shading), we then find two distinct field deformation topologies. *Turbulent-type* topology is seen during the MMS 3 encounter with the leading (green) and trailing (red) edges of the EDR. For this topology, the invariants track the magenta contour of triaxial deformations with eigenvalue ratios − 3:$-\,$1:4 ($R_s<0$) and 3:1:$-4$ ($R_s>0$), including the points near the magnetic null indicated by a yellow triangle. There is a departure from this contour, to *dimensionally reduced* topology when MMS 3 is within the EDR (blue), during which time it is confined to a line of constant $R_s$.

The turbulent type topology that is found in the magnetic field during the reconnection encounter coincides with that seen in the deformations of the velocity stream lines of *neutral fluid* turbulence on dissipative scales, obtained from the observed velocity gradient tensor^28,29^. As detailed in the Discussion below, this suggests a turbulent Euler-EMHD flow supported by the bulk, thermal electron population. Dimensionally reduced topology is seen in the departure from the magenta contour when MMS 3 is within the EDR (blue). During this time the magnetic field deformations plotted in panel (b) are instead confined to a line of constant $R=0$. This indicates that the variation of the magnetic field in one of the directions is fixed by the eigenvalues of the remaining two directions. The third eigenvalue is then fixed by $\lambda _3 = K(\lambda _1 \lambda _2)^{-1}=0$. It is a field line stretching which will render magnetic structures more sheet-like and will suppress the 3D EMHD supported turbulence. This is a path by which the curl-free, and perforce divergence free, magnetic field deformations can evolve toward a field null.

Anisotropy in the bulk electron thermal populations coincides with electric and magnetic field fluctuations at the edge of the EDR, whereas suprathermal electrons are seen within the EDR which is a region of persistently non-zero electric field (Fig. 2f,g). This is consistent with both simulations^43^ and observations^33,34^. Within the reconnection encounter, we have identified the topology of EMHD turbulent-type fluctuations. These turbulent fluctuations could quench the direct electric field acceleration of electrons to suprathermal energies. The overall effect would be to suppress electron acceleration throughout the reconnection encounter in regions where the topology tracks the turbulent type contour in Fig. 3c. Enhanced anisotropy of the bulk thermal electron population, and both electric and magnetic field fluctuations coincide with this turbulent-type topology of the magnetic field. Within the EDR, the topology is dimensionally reduced and departs from the signature of turbulence-type topology; this coincides with enhanced suprathermal electron flux (Fig. 2f) and isotropic bulk electrons.

**Figure 4:**
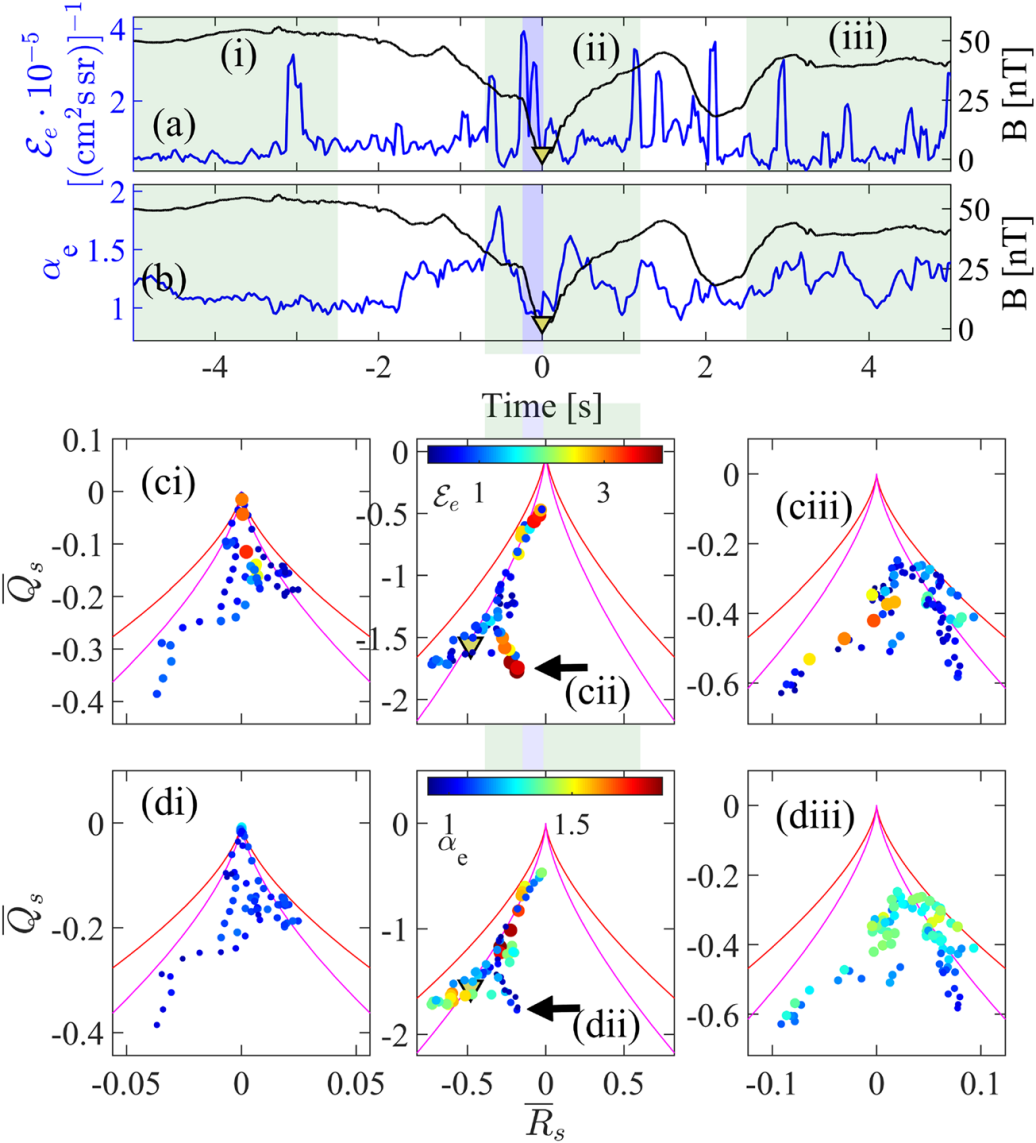
Electron energization and magnetic field topology. Observations at MMS 3 plotted as a function of time of (**a**): Differential electron flux integrated over energy range of 800 eV to $10^4$ eV and (**b**) electron temperature anisotropy. The shaded regions are selected time intervals before (i), during (ii) and after (iii) MMS 3 encounters the reconnection region at $t=0$; Panels (**c**) and (**d**) show these observations in the parameter space of the averaged topological invariants ${\overline{R}}_s$, ${\overline{Q}}_s$ of the magnetic field obtained from all four MMS spacecraft. Panels (ci)–(ciii) plot the integrated differential electron flux and panels (di)–(diii) plot the electron temperature anisotropy. The arrow indicates the same group of observations as indicated by blue markers in panels (**b**), (**c**) of Fig. 3. Solid red and magenta curves are the same as these described in Fig. 3, panel (**c**).

We verify this scenario in Fig. 4 where we directly map the observed electron suprathermal energy, $\mathcal {E}_e$, (integrated differential flux in the range of 800 eV to $10^4$ eV) and electron temperature anisotropy $\alpha _e$ onto the observed magnetic deformations in the same $R_s,Q_s$ space as Fig. 3c. The red contour again plots $\Delta _s=0$, the boundary of curl-free deformations of the magnetic field and the magenta contour indicates turbulent type topology identified in Fig. 3(c). The details of how these plots are constructed are given in the Methods. We consider three time intervals: (i) prior to the EDR encounter by any of the spacecraft, (ii) during the encounter (as in Fig. 3) and (iii) after the encounter with the EDR. These intervals are indicated on the MMS 3 time series in panels (a) and (b) of Fig. 4 where we show differential energy flux of suprathermal electrons, and the electron temperature anisotropy. Suprathermal electrons appear intermittently, and at the leading edge of the EDR rather than at the magnetic field null, as reported previously (eg^44^).

Panels (ci), (ciii) and (di), (diii) map quantities $\mathcal {E}_e$ and $\alpha _e$ onto $Q_s,R_s$ invariants space when all MMS spacecraft are outside of the EDR. The colour scale is common to all three panels in each row. We see that for time intervals (i) and (iii) the suprathermal energy is low and the temperature anisotropy is approximately 1, and there is no clear organization of energization with magnetic field line topology. During the reconnection encounter, time interval (ii), we again see the points track the turbulent like topology contour except during the EDR encounter where the topology is dimensionally reduced. The peak integrated energy flux $\mathcal {E}_e$ (cii) of suprathermal electrons coincides with dimensionally reduced field topology, whereas the peak of the thermal (bulk) electron temperature anisotropy $\alpha _e$ (dii) coincides with turbulent type magnetic field topology. The time at which the magnetic null point is encountered, indicated by the yellow triangle, does not coincide with a flux of energetic electrons. The field null is located at the transition between dimensionally reduced, and turbulent type topology. Figure 4 required temporal averaging of the topological invariants, they are low-pass filtered to match the timescale of the electron plasma observations (see Methods).

Particle distribution functions corroborate these findings and these are plotted in the SI. These plots show non-Maxwellian distributions for both the parallel and perpendicular velocity VDF. The parallel velocity VDF show anisotropic heating at the time when the peak in the electron temperature anisotropy is found in Fig. 2 panel (g). The perpendicular velocity VDF does not change significantly until the MMS 3 is in the EDR, when it shows both an shift in its mean and elevated tails.

## Discussion and conclusions

High cadence, four spacecraft observations have allowed us to directly determine the topology of the magnetic field in-situ within a reconnection site. The magnetic field gradients, and the corresponding magnetic topology, are directly spatially resolved on the scale of the spacecraft separation. However, how the topology varies spatially is indirectly captured on a much finer spatial scale with the fast sampling in time of the magnetic field simultaneously at all four spacecraft. We resolve the trajectory in the invariant space that identifies this topology on the fast sampling timescale. Thus we can directly compare how the magnetic field topology is changing with signatures of electron heating and acceleration which are also available at high time cadence.

Previously, mechanisms proposed for turbulent reconnection^6–8,10–13^, are processes that act across, and encompass, multiple reconnection sites. We have identified the topological signature of fluid-like turbulence within a single reconnection site. We find that the topology of turbulent deformation of magnetic field lines directly orders electron acceleration. Fluid-like turbulent topology is found where suprathermal electrons are absent; when the topology transitions to reduced dimensionality and is closer to sheet-like in the electron diffusion region (EDR), suprathermal electrons are encountered. This suggest that the magnetic topological features that we have identified are thus an integral part of how electrons are accelerated by reconnection.

The topology of turbulent deformation of magnetic field lines that we have identified coincides with that seen in an ideal turbulent hydrodynamic flow. The correspondence between magnetic field rotation-free deformations and the behaviour of the dissipative structures in HD turbulence can be understood as follows. The spacecraft tetrahedron spans scales on which the ions are effectively demagnetised, so EMHD will apply. For a near-collisionless plasma, considered here, the magnetic field is effectively frozen into the electron fluid^45^, and if we can neglect electron inertia, the induction equation then becomes:

1 $$\begin{aligned} \frac{\partial \varvec{B}}{\partial t}=\nabla \times (\varvec{v}_e \times \varvec{B}), \end{aligned}$$

where $\varvec{v}_e$ is the velocity field of the electrons. We then expect the topology of fluctuations in the magnetic field, and its scaling properties, to track that of the electron fluid. When the scales of the fluctuations become very short, on scales $d \ll d_e$, where the electron vorticity $w_e\gg j/d_e$, EMHD reduces to the Euler equation for the electron fluid velocity field^7^. The topology of this turbulent electron flow will then be identical to ideal hydrodynamic flow, as they are both constrained by the same Euler equation. It is the clear time-ordered flow of the invariants in their phase space near the EDR (Fig. 3c) that supports this hypothesis. Away from the encounter, we do not detect such ordered flow and the invariants show more random behaviour, indicative of sampling uncorrelated structures with different topology. Taking the maximal electron fluid velocity of $\sim \!800$ km s$^{-1}$ as measured by the MMS 3 at the EDR, and the sampling frequency of the magnetic field at 8192 Hz, we obtain the corresponding spatial scale of the temporal measurement to be $d \approx \! 0.1$ km, as compared to the electron inertial length $d_e = 1.2$ km.

An alternative scenario is fast self-similar current sheet fragmentation process via finite length plasmoid production. The lack of lasting elliptic magnetic field line features, indicative of plasmoids, within the encounter suggests that this hypothesis is less likely.

Our results provide direct observational constraints on the topology that can discriminate these mechanisms. In this first study we have analysed one well-sampled reconnection event. Routine computation of field topological invariants in the studies of reconnection, both in observations, and simulations, would establish whether this behaviour is ubiquitous, or indeed, universal, or under what conditions it occurs.

## Methods

### Reconnection event overview

There are several quantities which indicate an encounter of the spacecraft with the EDR and these are shown in panels (a)–(h) of Fig. 2 in the main text. The extent of the EDR is marked by the blue shaded region near $t=0$ for each panel. Panel (a) of Fig. 2 shows the decrease in magnetic field magnitude near the X-point crossed at 07:43:30.488 and this crossing defines the epoch time $t=0$. We use merged FGM^46^ and SCM^47^ instrument magnetic field data with the sampling frequency 8192 Hz. The blue trace shows one electric field component, $E_y$ in GSE coordinates, obtained from the EDP instrument^48,49^ with sampling frequency of 8192 Hz. A secular negative excursion of this field component in the vicinity of $t=0$ defines the EDR^35^. The magnetic and electric field magnitude and component fluctuations, shown in panels (b) and (c), are calculated using a band pass filter with frequency range 64–256 Hz. Panel (d) shows the expected behaviour of the ion and electron velocities, normalised to the downstream Alfvén speed, $v_{A,ds} = B_{ds} / \sqrt{\mu _0 n_{p,ds} m_p}$, with electrons accelerated by a factor of few Alfvén speeds within a narrow region within the EDR. The electron velocity distributions and their moments are from FPI-DEM instrument^50^ with sampling frequency of 33 Hz. Panel (e) shows the power density transferred between particles and fields, $P^m_e = \varvec{j} \cdot \varvec{E}_e$, where $\varvec{E}_e$ is the electric field in the electron frame of reference^51^, $\varvec{E}_e = \varvec{E} + \varvec{v}_e \times \varvec{B}$ and the current density obtained from particle data, $\varvec{j}=q n_e(\varvec{v}_i - \varvec{v}_e)$. A positive $P^m_e$ indicates a loss of electromagnetic energy, which is converted to macroscopic electron flows. Where particles give their energy back to the electromagnetic fluctuations $P^m_e$ is negative. Overall we observe a net transfer of fields’ energy to particles in the shaded region. The MMS mission measures the differential electron flux as a function of energy and pitch angle. Panel (f) shows the omni-directional electron flux integrated over a range of energies, $\mathcal {E}_e$, defined as

2 $$\begin{aligned} \mathcal {E}_e = (E_1 - E_0)^{-1} \int _{E_0}^{E_1} \Phi _e dE, \end{aligned}$$

where $E_0=800$ eV and $E_1=10^4$ eV are the chosen minimum and the maximum energy values and $\Phi _e$ is the differential electron flux in the units of [(cm$^2$ s sr)$^{-1}$]. We observe a modest increase in the electron flux for higher energies, and critically the maximum is not at the suspected X-line position $t=0$. The reversal of the magnetic field near the X-point, shown in panel (a), must generate an electric field^52^ which, at least in principle, can accelerate electrons to extremely large energies. Three-dimensional PIC simulation shows that such direct acceleration is not efficient when the guide field is weak, as it is in our case^53^. On larger scales, the fast release of the local magnetic field tension re-configures the field and these changes propagate at approximately Alfvén speed. Away from the X-point particles are still magnetized and are accelerated by this motion of the newly generated magnetic field lines. This should lead to increase in the electron temperature anisotropy, $\alpha _e$, which is shown in the panel (g). These quantities are also plotted in Fig. 4a,b. In order to project the quantities $\mathcal {E}_e$ and $\alpha _e$ onto the phase space of the magnetic field topological invariants ($Q_s$, $R_s$), we average the invariants between each two consecutive electron measurements (over approximately 248 magnetic data points) obtaining integrated electron flux and the electron temperature anisotropy associated with an averaged value of invariants (${\overline{R}}_s$ and ${\overline{Q}}_s)$), $\mathcal {E}_e({\overline{R}}_s,{\overline{Q}}_s)$ and $\alpha _e({\overline{R}}_s, {\overline{Q}}_s)$.

### Magnetic field gradient tensor

We have obtained the MFGT using two approaches, based on the Taylor expansion of the magnetic field at a point which retains only linear terms in the spacecraft position. A magnetic field vector is then written as

3 $$\begin{aligned} \varvec{B}(\varvec{r}) = \varvec{B}(\varvec{r}_0) + \nabla \varvec{B} (\varvec{r}-\varvec{r}_0), \end{aligned}$$

where $\varvec{r}$ represents spacecraft position, in our case, in GSE coordinates.

The first approach uses differences between spacecraft pairs and expresses the relation between magnetic field increment and spacecraft separation vectors as a matrix equation^39,40^. Defined with respect to the centroid, $\varvec{\rho }_{1}\!=\!(\varvec{r}_1-\varvec{r}_2)/\sqrt{2}$, $\varvec{\rho }_{2}\!=\!(\varvec{r}_1+\varvec{r}_2-2\varvec{r}_3)/\sqrt{6}$, and $\varvec{\rho }_{3}\!=\!(\varvec{r}_1+\varvec{r}_2+\varvec{r}_3-3\varvec{r}_4)/\sqrt{12}$. Similarly, $\varvec{b}_{1}\!=\!(\varvec{B}_1-\varvec{B}_2)/\sqrt{2}$, $\varvec{b}_{2}\!=\!(\varvec{B}_1+\varvec{B}_2-2\varvec{B}_3)/\sqrt{6}$, $\varvec{b}_{3}\!=\!(\varvec{B}_1+\varvec{B}_2+\varvec{B}_3-3\varvec{B}_4)/\sqrt{12}$, and $\varvec{B}_s$ is the magnetic field vector at spacecraft *s*. Inverting the relation $\textbf{b}\!=\!\varvec{A}\varvec{\rho }$ with the constraint $\nabla \cdot \varvec{b}=0$ gives the tensor elements $M_{ij}\!=\!(\varvec{b}\varvec{\rho }^{-1})_{ij}-\delta _{ij}{\text {tr}} \left( \varvec{b} \varvec{\rho }^{-1} \right) /3$. For the interval considered here, this method produced MFGTs with the average trace values of $\sim \! 1 \times 10^{-4}$, while appropriately normalised tensor invariants of interest reached values of orders $0.1-1$.

The second approach is based on nonlinear least squares fits of the polynomial (3) to the measured magnetic field^41^. Note that this method has 12 parameters, 3 components of $\varvec{B}(\varvec{r}_0)$ and 9 elements of the MFGT. Given 4 spacecraft, each measuring 3 components of the magnetic field vector, we have 12 equations to be solved, thus the problem is well-posed. We have used Matlab’s lsqnonlin function to perform the least squares fit with the constraint of the divergence-free solution. This method produced an MFGT with trace no larger then $\sim \! 1 \times 10^{-14}$, while appropriately normalised tensor invariants were still of order 0.1–1. Recently, second order methods have been proposed, which use particle currents at each spacecraft in addition to magnetic field data^41,42^. These methods may give more accurate results when the current is strong, which is not the case in our interval.

**Figure 5:**
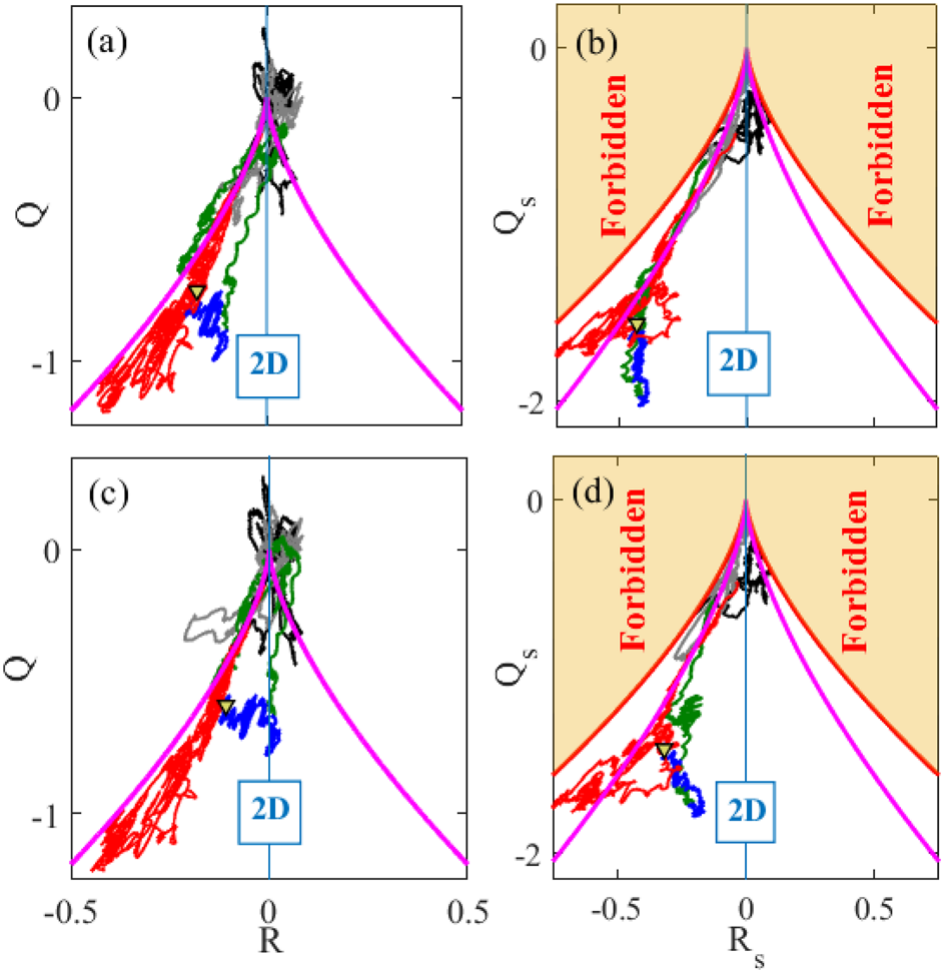
Comparison of results obtained by different methods. Panels (**a**) and (**b**) plot the space of invariants (R, Q) and (R$_s$, Q$_s$), respectively, of the MFGT obtained by direct matrix inversion^39,40^. Panels (**c**) and (**d**) shows the same results obtained from the nonlinear least squares method^41^, used in the main text.

Figure 5 shows differences between the phase spaces (R, Q) and (R$_s$, Q$_s$) obtained by these two different approaches. It is clear that the overall flow of invariants in their phase space is the same, but there are small differences in how the invariants approach 2D line of $R=0$ in panels (a) and (c). The nonlinear least squares method shows that the magnetic field topology has reduced dimensionality at the EDR, that is, the magnetic field variation in one of the direction is negligible. The dynamics of the points within the EDR, those shown in blue, is also different. The matrix inversion method shows almost no variation in the values of R, while the least squares method does show some variations.

The topological invariants, *R* and *Q*, of this tensor characterise the magnetic field line topology^23,25,26^. Given three eigenvalues of the MFGT, $\lambda _1$, $\lambda _2$ and $\lambda _3$, the main invariants can be computed from the following expressions^54^:

4 $$\begin{aligned} {\tilde{Q}} = -\frac{1}{2}{\text {tr}}(\varvec{M}^2) = \lambda _1 \lambda _2 + \lambda _1 \lambda _3 + \lambda _2 \lambda _3,\, {\tilde{R}} = -\frac{1}{3}{\text {tr}}(\varvec{M}^3) = - \lambda _1 \lambda _2 \lambda _3. \end{aligned}$$

The symmetric part of $\varvec{M}$, $\varvec{S}=(\varvec{M}+\varvec{M}^T)/2$, relates to a rate of strain of magnetic field lines, that is, it accounts for all deformations of the magnetic field topology which are not related to a current. The invariants of $\varvec{S}$ will be indicated by $R_s$, $Q_s$. The antisymmetric current density rate tensor $\varvec{J}=(\varvec{M}-\varvec{M}^T)/2$ relates to current density, $j_k\!=\!\epsilon _{ijk} J_{ij}$ via the Faraday law $\nabla \times \varvec{B}=\mu _0 \varvec{j}$. All invariants are normalised by the power in $j^2$, for example, we infer the topology from $Q(t) \!=\! \tilde{Q}(t)/\langle j^2 \rangle$ and $R(t) \!=\! \tilde{R}(t)/\langle j^2 \rangle ^{3/2}$, where $\langle j^2 \rangle$ is the time average.

The sign of the quantity $\Delta = (27/4)R^2+Q^3$ defines two regions in the (*R*, *Q*) plane. Invariants that give $\Delta >0$ indicate elliptic magnetic field lines, while for $\Delta <0$ the field lines are hyperbolic, forming a multi-pole separatrix structure consistent with 3D reconnection (3D X-line). When $\Delta >0$, two eigenvalues of $\varvec{M}$ are complex and one is real, indicative of the magnetic flux rope topology that generalises a two-dimensional O-point in three dimensions. We note that quasi-2D flux ropes with a negligible magnetic field variation along the structure on the scale of the spacecraft tetrahedron have $R\! \sim \! 0$, while finite length, self-contained structures, such as three-dimensional plasmoids, have $|R| \gg 0$. For $\Delta <0$ all three eigenvalues are real and such hyperbolic magnetic field line topology is the 3D generalisation of a 2D X-point. The sign of *R* discriminates between two different directions of the magnetic field vector for each topology.

The deformation tensor invariants are defined in a similar way. If $\lambda ^s_1$, $\lambda ^s_2$, and $\lambda ^s_3$ are the eigenvalues of tensor $\varvec{S}$, then

5 $$\begin{aligned} {\tilde{Q}}_s = -\frac{1}{2}{\text {tr}}(\varvec{S}^2) = \lambda ^s_1 \lambda ^s_2 + \lambda ^s_1 \lambda ^s_3 + \lambda ^s_2 \lambda ^s_3,\, {\tilde{R}}_s = -\frac{1}{3}{\text {tr}}(\varvec{S}^3) = -\lambda ^s_1 \lambda ^s_2 \lambda ^s_3. \end{aligned}$$

Applying the same normalisation as was used for $\tilde{R}$ and $\tilde{Q}^3$, we obtain invariants $R_s$, $Q_s$. Recall that the eigenvalues of a symmetric tensor tensor must be real. This reduces the available phase space of the invariants $R_s$, $Q_s$, to a region $\Delta _s = (27/4)R_s^2+Q_s^3 \le 0$. The red contour in Fig. 3c is the boundary of this available phase space, and corresponds to the axis-symmetric contraction ($R_s<0$) with the eigenvalue ratios of 2:$-1$:$-1$, or axis-symmetric expansion ($R_s>0$) with the eigenvalue ratios 1:1:$-2$. The magenta contour in this panel coincides with the triaxial deformations given by eigenvalue ratios $-3$:$-1$:4 ($R_s<0$) and 3:1:$-4$ ($R_s>0$). The eigenvalue ratio 3:1:$-4$ ($R_s>0$) has been found to characterise the deformations of the velocity field gradient tensor in a three dimensional hydrodynamic turbulent flow^28,29^. The opposite sign of this ratio found in our work reflects a particular magnetic field polarity in the reconnection region.

### Supplementary Information

Supplementary Information.

## Data Availability

The datasets generated and/or analysed during the current study are available in the MMS Science Data Center repository, https://lasp.colorado.edu/mms/sdc/public/.

## References

[1] Birn J, Priest ER (2007). Reconnection of Magnetic Fields: Magnetohydrodynamics and Collisionless Theory and Observations.

[2] Krommes JA (2002). Fundamental statistical descriptions of plasma turbulence in magnetic fields. Phys. Rep..

[3] Matthaeus WH, Velli M (2011). Who needs turbulence?. Space Sci. Rev..

[4] Lemoine M (2021). Particle acceleration in strong MHD turbulence. Phys. Rev. D.

[5] Egedal J, Daughton W, Le A (2012). Large-scale electron acceleration by parallel electric fields during magnetic reconnection. Nat. Phys..

[6] Lazarian A, Eyink G, Vishniac E, Kowal G (2015). Turbulent reconnection and its implications. Philos. Trans. R. Soc. A Math. Phys. Eng. Sci..

[7] Biskamp D, Schwarz E, Drake JF (1997). Two-fluid theory of collisionless magnetic reconnection. Phys. Plasmas.

[8] Linton MG, Dahlburg RB, Antiochos SK (2001). Reconnection of twisted flux tubes as a function of contact angle. Astrophys. J..

[9] Karimabadi H, Roytershteyn V, Wan M, Matthaeus WH, Daughton W, Wu P, Shay M, Loring B, Borovsky J, Leonardis E, Chapman SC, Nakamura TKM (2013). Coherent structures, intermittent turbulence and dissipation in high-temperature plasmas. Phys. Plasmas.

[10] Zhou M, Wu DH, Loureiro NF, Uzdensky DA (2021). Statistical description of coalescing magnetic islands via magnetic reconnection. J. Plasma Phys..

[11] Drake JF, Swisdak M, Schoeffler KM, Rogers BN, Kobayashi S (2006). Formation of secondary islands during magnetic reconnection. Geophys. Res. Lett..

[12] Daughton W, Scudder J, Karimabadi H (2006). Fully kinetic simulations of undriven magnetic reconnection with open boundary conditions. Phys. Plasmas.

[13] Lu S, Angelopoulos V, Artemyev AV, Pritchett PL, Sun WJ, Slavin JA (2020). Particle-in-cell simulations of secondary magnetic islands: Ion-scale flux ropes and plasmoids. Astrophys. J..

[14] Del Sarto D, Califano F, Pegoraro F (2003). Secondary instabilities and vortex formation in collisionless-fluid magnetic reconnection. Phys. Rev. Lett..

[15] Eriksson S, Newman DL, Lapenta G, Angelopoulos V (2014). On the signatures of magnetic islands and multiple X-lines in the solar wind as observed by ARTEMIS and WIND. Plasma Phys. Control. Fusion.

[16] Zhong ZH, Zhou M, Liu YH, Deng XH, Tang RX, Graham DB, Song LJ, Man HY, Pang Y, Khotyaintsev YV (2022). Stacked electron diffusion regions and electron Kelvin–Helmholtz vortices within the ion diffusion region of collisionless magnetic reconnection. Astrophys. J. Lett..

[17] Strauss HR (1988). Turbulent reconnection. Astrophys. J..

[18] Eastwood JP, Phan TD, Bale SD, Tjulin A (2009). Observations of turbulence generated by magnetic reconnection. Phys. Rev. Lett..

[19] Eastwood JP, Shay MA, Phan TD, Oieroset M (2010). Asymmetry of the ion diffusion region Hall electric and magnetic fields during guide field reconnection: Observations and comparison with simulations. Phys. Rev. Lett..

[20] Fuselier SA, Vines SK, Burch JL, Petrinec SM, Trattner KJ, Cassak PA, Chen LJ, Ergun RE, Eriksson S, Giles BL, Graham DB (2017). Large-scale characteristics of reconnection diffusion regions and associated magnetopause crossings observed by MMS. J. Geophys. Res. Space Phys..

[21] Daughton W, Roytershteyn V, Karimabadi H, Yin L, Albright BJ, Bergen B, Bowers KJ (2011). Role of electron physics in the development of turbulent magnetic reconnection in collisionless plasmas. Nat. Phys..

[22] Leonardis E, Chapman SC, Daughton W, Roytershteyn V, Karimabadi H (2013). Identification of intermittent multi-fractal turbulence in fully kinetic simulations of magnetic reconnection. Phys. Rev. Lett..

[23] Dallas V, Alexakis A (2013). Structures and dynamics of small scales in decaying magnetohydrodynamic turbulence. Phys. Fluids.

[24] Fu HS, Vaivads A, Khotyaintsev YV, Olshevsky V, André M, Cao JB, Huang SY, Retino A, Lapenta G (2015). How to find magnetic nulls and reconstruct field topology with MMS data?. J. Geophys. Res. Space Phys..

[25] Quattrociocchi V, Consolini G, Marcucci MF, Materassi M (2019). On geometrical invariants of the magnetic field gradient tensor in turbulent space plasmas: Scale variability in the inertial range. Astrophys. J..

[26] Hnat B, Chapman SC, Watkins NW (2021). Magnetic topology of actively evolving and passively convecting structures in the turbulent solar wind. Phys. Rev. Lett..

[27] Greene JM (1988). Geometrical properties of three-dimensional reconnecting magnetic fields with nulls. J. Geophys. Res. Space Phys..

[28] Ashurst WT, Kerstein AR, Kerr RM, Gibson CH (1987). Alignment of vorticity and scalar gradient with strain rate in simulated Navier–Stokes turbulence. Phys. Fluids.

[29] Tsinober A, Kit E, Dracos T (1992). Experimental investigation of the field of velocity gradients in turbulent flows. J. Fluid Mech..

[30] Lin RP (2011). Energy release and particle acceleration in flares: Summary and future prospects. Space Sci. Rev..

[31] Zhang Q, Guo F, Daughton W, Li H, Li X (2021). Efficient nonthermal ion and electron acceleration enabled by the flux-rope kink instability in 3D nonrelativistic magnetic reconnection. Phys. Rev. Lett..

[32] Øieroset M, Lin RP, Phan TD, Larson DE, Bale SD (2002). Evidence for electron acceleration up to 300 keV in the magnetic reconnection diffusion. Phys. Rev. Lett..

[33] Imada S, Hoshino M, Mukai T (2005). Average profiles of energetic and thermal electrons in the magnetotail reconnection regions. Geophys. Res. Lett..

[34] Phan TD, Eastwood JP, Shay MA, Drake JF, Sonnerup BU, Fujimoto M, Cassak PA, Oieroset M, Burch JL, Torbert RB, Rager AC (2018). Electron magnetic reconnection without ion coupling in Earth’s turbulent magnetosheath. Nature.

[35] Chen LJ, Hesse M, Wang S, Gershman D, Ergun R, Pollock C, Torbert R, Bessho N, Daughton W, Dorelli J, Giles B (2016). Electron energization and mixing observed by MMS in the vicinity of an electron diffusion region during magnetopause reconnection. Geophys. Res. Lett..

[36] Fuselier SA, Lewis WS, Schiff C, Ergun R, Burch JL, Petrinec SM, Trattner KJ (2016). Magnetospheric multiscale science mission profile and operations. Space Sci. Rev..

[37] Cao D, Fu HS, Cao JB, Wang TY, Graham DB, Chen ZZ, Peng FZ, Huang SY, Khotyaintsev YV, André M, Russell CT (2017). MMS observations of whistler waves in electron diffusion region. Geophys. Res. Lett..

[38] Zhong ZH, Tang RX, Zhou M, Deng XH, Pang Y, Paterson WR, Giles BL, Burch JL, Tobert RB, Ergun RE, Khotyaintsev YV (2018). Evidence for secondary flux rope generated by the electron Kelvin–Helmholtz instability in a magnetic reconnection diffusion region. Phys. Rev. Lett..

[39] Paschmann, G. & Daly, P.W. (Eds). Analysis methods for multi-spacecraft data. ISSI Scientific Report SR-001 (1998)

[40] Chertkov M, Pumir A, Shraiman BI (1999). Lagrangian tetrad dynamics and the phenomenology of turbulence. Phys. Fluids.

[41] Denton RE (2020). Polynomial reconstruction of the reconnection magnetic field observed by multiple spacecraft. J. Geophys. Res. Space Phys..

[42] Torbert RB, Dors I, Argall MR, Genestreti KJ, Burch JL, Farrugia CJ (2020). A new method of 3-D magnetic field reconstruction. Geophys. Res. Lett..

[43] Lapenta G, Berchem J, El Alaoui M, Walker R (2020). Turbulent energization of electron power law tails during magnetic reconnection. Phys. Rev. Lett..

[44] Haynes CT, Burgess D, Camporeale E (2014). Reconnection and electron temperature anisotropy in sub-proton scale plasma turbulence. Astrophys. J..

[45] Lighthill MJ (1960). Studies on magneto-hydrodynamic waves and other anisotropic wave motions. Philos. Trans. R. Soc. Lond. Ser. A Math. Phys. Sci..

[46] Russell CT, Anderson BJ, Baumjohann W, Bromund KR, Dearborn D, Fischer D, Le G, Leinweber HK, Leneman D, Magnes W, Means JD, Moldwin MB, Nakamura R, Pierce D, Plaschke F, Rowe KM, Slavin JA, Strangeway RJ, Torbert R, Hagen C, Jernej I, Valavanoglou A, Richter I (2016). The magnetospheric multiscale magnetometers. Space Sci. Rev..

[47] Le Contel O, Leroy P, Roux A, Coillot C, Alison D, Bouabdellah A, Mirioni L, Meslier L, Galic A, Vassal MC, Torbert RB (2016). The search-coil magnetometer for MMS. Space Sci. Rev..

[48] Ergun RE, Tucker S, Westfall J, Goodrich KA, Malaspina DM, Summers D, Wallace J, Karlsson M, Mack J, Brennan N, Pyke B, Withnell P, Torbert R, Macri J, Rau D, Dors I, Needell J, Lindqvist PA, Olsson G, Cully CM (2016). The axial double probe and fields signal processing for the MMS mission. Space Sci. Rev..

[49] Lindqvist PA, Olsson G, Torbert RB, King B, Granoff M, Rau D, Needell G, Turco S, Dors I, Beckman P, Macri J, Frost C, Salwen J, Eriksson A, Ahlen L, Khotyaintsev YV, Porter J, Lappalainen K, Ergun RE, Wermeer W, Tucker S (2016). The spin-plane double probe electric field instrument for MMS. Space Sci. Rev..

[50] Pollock C, Moore T, Jacques A, Burch J, Gliese U, Saito Y, Omoto T, Avanov L, Barrie A, Coffey V, Dorelli J, Gershman D, Giles B, Rosnack T, Salo C, Yokota S, Adrian M, Aoustin C, Auletti C, Aung S, Bigio V, Cao N, Chandler M, Chornay D, Christian K, Clark G, Collinson G, Corris T, De Los Santos A, Devlin R, Diaz T, Dickerson T, Dickson C, Diekmann A, Diggs F, Duncan C, Figueroa-Vinas A, Firman C, Freeman M, Galassi N, Garcia K, Goodhart G, Guererro D, Hageman J, Hanley J, Hemminger E, Holland M, Hutchins M, James T, Jones W, Kreisler S, Kujawski J, Lavu V, Lobell J, LeCompte E, Lukemire A, MacDonald E, Mariano A, Mukai T, Narayanan K, Nguyan Q, Onizuka M, Paterson W, Persyn S, Piepgrass B, Cheney F, Rager A, Raghuram T, Ramil A, Reichenthal L, Rodriguez H, Rouzaud J, Rucker A, Saito Y, Samara M, Sauvaud J-A, Schuster D, Shappirio M, Shelton K, Sher D, Smith D, Smith K, Smith S, Steinfeld D, Szymkiewicz R, Tanimoto K, Taylor J, Tucker C, Tull K, Uhl A, Vloet J, Walpole P, Weidner S, White D, Winkert G, Yeh P-S, Zeuch M (2016). Fast plasma investigation for magnetospheric multiscale. Space Sci. Rev..

[51] Zenitani S, Hesse M, Klimas A, Kuznetsova M (2011). New measure of the dissipation region in collisionless magnetic reconnection. Phys. Rev. Lett..

[52] Pritchett PL, Mozer FS (2009). The magnetic field reconnection site and dissipation region. Phys. Plasmas.

[53] Dahlin JT, Drake JF, Swisdak M (2017). The role of three-dimensional transport in driving enhanced electron acceleration during magnetic reconnection. Phys. Plasmas.

[54] Meneveau C (2011). Lagrangian dynamics and models of the velocity gradient tensor in turbulent flows. Annu. Rev. Fluid Mech..

[55] Fu HS, Khotyaintsev YV, Vaivads A, Retinò A, André M (2013). Energetic electron acceleration by unsteady magnetic reconnection. Nat. Phys..

