# Supplementary material for: Topology of turbulence within collisionless plasma reconnection
Source: Sci Rep. 2023 Oct 31;13:18665. doi: 10.1038/s41598-023-45650-x (PMC10618222; doi:10.1038/s41598-023-45650-x)
Supplement: Supplementary file 1 — Supplementary Information. [file 41598_2023_45650_MOESM1_ESM.pdf]

Supplementary Information: Topology of turbulence within collisionless plasma reconnection

B. Hnat[1], S. C. Chapman[1,2,3], and N. W. Watkins[1,4,5]

[1]Centre for Fusion Space and Astrophysics, Physics Department, University of Warwick, Coventry, UK
[2]Department of Mathematics and Statistics, University of Tromso, Norway
[3]International Space Science Institute, Bern, Switzerland
[4]Grantham Research Institute on Climate Change and the Environment, London School of Economics and Political Science, Houghton Street, London, UK
[5]School of Engineering and Innovation, The Open University, Milton Keynes, UK

Contents of Supplementary Information

1. Supplementary Note 1, Supplementary Figure 1
2. Supplementary Note 2, Supplementary Figure 2
3. Supplementary Note 3, Supplementary Figure 3
4. Supplementary Note 4, Supplementary Figure 4

Supplementary Note 1

The estimated gradient is obtained over the volume of the four spacecraft tetrahedron, thus in principle, it would be optimal to represent all plasma quantities as averages centred on the spacecraft tetrahedron centroid. However, since the spacecraft experience quite different conditions at any given time, such averaging may not be representative of the physics of the reconnection region, especially the Electron Diffusion Region (EDR). The MMS 3 spacecraft shows the clearest signatures of crossing the EDR and so we have focused on electron data from MMS3. During the reconnection event, MMS 3 is located between MMS4 and MMS 1 and 2, and thus is closest to the centroid of the spacecraft tetrahedron. Supplementary figure 1 plots, for each of the four spacecraft, magnetic field strength, electron velocity M component in the event LMN coordinates normalised to the local Alfvén speed, and electron temperature anisotropy at each spacecraft location. In each set of panels, the top panel plots the magnetic field magnitude at all four spacecraft and the Vertical lines indicate the minima of the magnetic field strength at each spacecraft, for MMS 4 where there are multiple minima, we have used the zero-crossing of the B_L component. The left set of panels splits out these magnetic field magnitude traces and shows a clear time sequence with MMS 4 detecting magnetic null first, followed by MMS 3 and then MMS 1 and 2, which detect the minimum of magnetic strength at nearly the same time. The colored vertical lines, which indicate the magnetic null encounter times, are then overlaid on the M component of the electron velocity from all four spacecraft (center column) and the electron temperature anisotropy from all four spacecraft (right column). Although not all features are seen at all four spacecraft, MMS4 encounters the electron velocity enhancement before MMS3 (center column), and MMS 1 and 2 encounter the peak-trough-peak signature in the electron anisotropy after MMS3 (right column). This is consistent with MMS 3 sampling plasma with similar properties to that at the four spacecraft tetrahedron centroid in time.

Supplementary Note 2

We plot velocity distribution functions (VDFs) to identify the contribution of suprathermal electrons to the maxima of the integrated flux shown in figure 2, panel(f) of the main paper. Enhanced flux can arise from either suprathermal tails broadening of the core of the distribution, to discriminate these, we have examined the VDFs at different times in the reconnection encounter. Supplementary Figure 2 directly compares the electron VDFs at MMS3 at three times: at 07:43:28.024 when MMS 3 is outside the reconnection region, at 07:43:29.014 when MMS3 observed increased electron temperature anisotropy (first peak of A_e in panel (g) of figure 2 of the main paper), and at 07:43:30.394 when the MMS3 is within the EDR. In supplementary figure 2 the

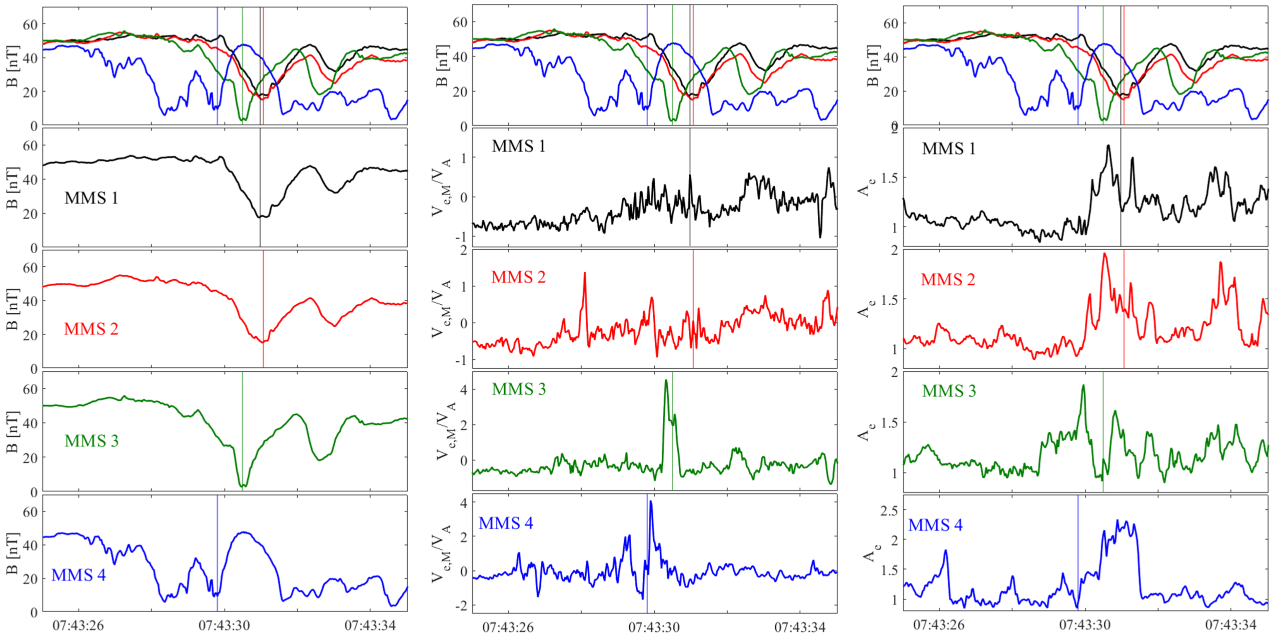

Supplementary Figure 1. Timing of the observations from all four spacecraft. Left column: magnetic field magnitude, center column: electron M component velocity normalised to the downstream Alfvén speed, right column: electron temperature anisotropy. Top panel in each column plots magnetic field magnitude at each of the four spacecraft. Vertical lines indicate the minima of the magnetic field strength at each spacecraft. Throughout the figure black traces refer to MMS 1, red to MMS2, green to MMS 3 and blue corresponds to MMS 4.

left-hand panels (a)-(c) plot the distributions for the velocity parallel with respect to the local magnetic field, while right-hand side panels (d)-(f) show distributions of the velocity in the direction perpendicular to the magnetic field. We obtain the least squares fit of a Maxwellian distribution to the core part of the distributions shown in (a) and (d), that is, when MMS3 is outside the reconnection region, these are plotted as solid black lines. The panel (a) VDF (green) and its Maxwellian fit (black) are then overplotted on figures (b) and (c), and in a similar manner, the panel (d) VDF (green) and its Maxwellain fit (black) are then overplotted on figures (e) and (f), so that we can see how the distribution evolves.

Both the parallel and perpendicular VDFs have non-Maxwellian distributions. Comparing panel (a) and (b), the parallel velocity VDFs show anisotropic heating at the time when the peak in the electron temperature anisotropy is found (see figure 2 panel (g) of the main paper). Comparing panels (d), (e) and (f), the perpendicular velocity VDF does not change significantly until MMS 3 is in the EDR, in panel (f) we see both a shift in the VDF mean and elevated tails. These VDF features are consistent with the observed increased energetic electron flux identifed in figure 2, panel (f) of the main paper being associated with suprathermal electrons at the edge and within the EDR.

Supplementary Note 3

In the manuscript, the magnetic field gradient tensor and its invariants are constructed from merged FGM/SCM data with sampling rate of 8192 samples per second. Figure 4 of the manuscript is then generated by re-sampling the invariants to the time resolution of the electron data (33 samples per second). We have confirmed that this is equivalent to constructing the invariants based on down-sampled magnetic field data. To do this, we have applied a method suggested in [Torbert et al., 2020] and down-sampled the magnetic field to a time cadence of 6 samples per second which approximately corresponds to the propagation time across the spacecraft separation based upon the bulk flow velocity. This is equal to the ion plasma instrument sampling rate. Figure 3 shows the main invariants obtained from down-sampled magnetic field as cyan squares, overplotted on the invariants from the combined FGM/SCM data.

Supplementary Note 4

Supplementary figure 4 shows the construction of the merged FGM/SCM dataset.

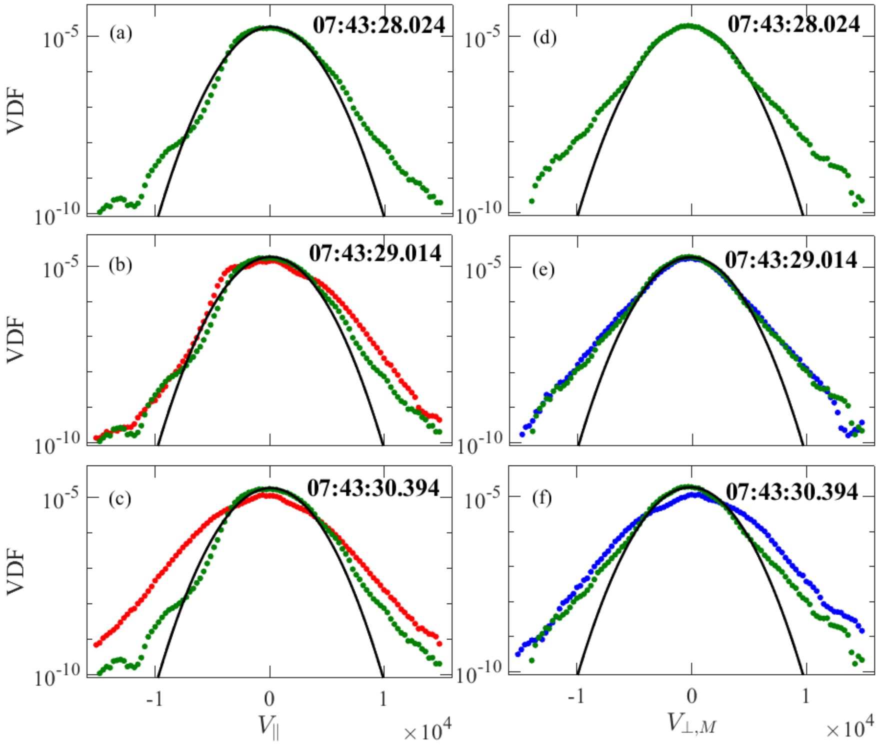

Supplementary Figure 2. MMS3 velocity distribution functions at three different times. Electron VDFs at MMS3 at three times: (a,d) at 07:43:28.024 when MMS 3 is outside the reconnection region, (b,e) at 07:43:29.014 when MMS3 observed increased electron temperature anisotropy (first peak of A_e in panel (g) of figure 2 of the main paper), and (c,f) at 07:43:30.394 when the MMS3 is within the EDR. Panels (a)-(c) and (d)-(f) show VDFs in the parallel and perpendicular direction with respect to the magnetic field, respectively. A least squares fit of a Maxwellian distribution to the core part of the distributions shown in (a) and (d), that is, when MMS3 is outside the reconnection region, is plotted as solid black lines. The panel (a) VDF (green) and its Maxwellian fit (black) are then overplotted on figures (b) and (c) and in a similar manner, the panel (d) VDF (green) and its Maxwellian fit (black) are then overplotted on figures (e) and (f), where red and blue indicate parallel and perpendicular velocity VDFs respectively.

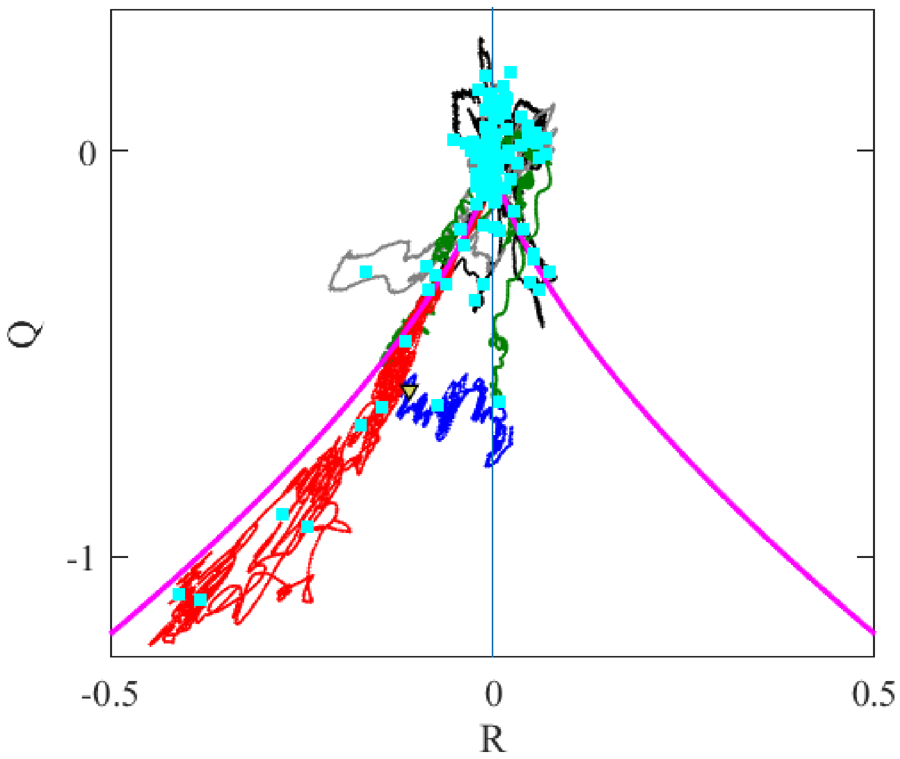

Supplementary Figure 3. Magnetic field gradient tensor invariants Q, R. Grey, green, blue, red and black traces are these from figure 3 of the manuscript and were obtained from the merged FGM/SCM magnetic data. Cyan square symbols show invariants constructed from the magnetic field down-sampled to ion sampling rate of 6 samples per second.

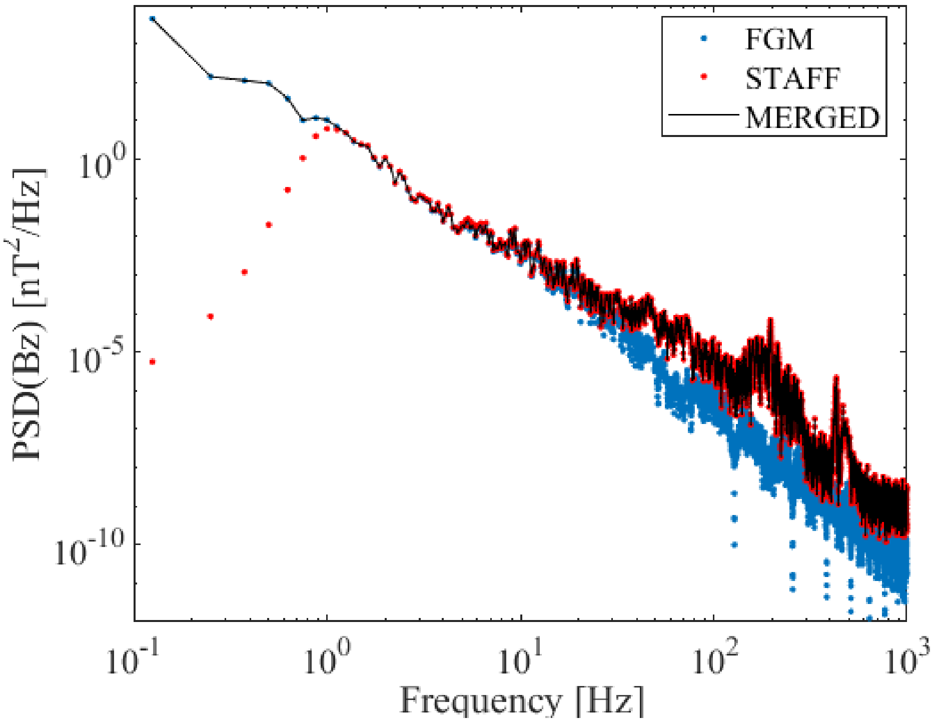

Supplementary Figure 4. Power spectral density of the GSE B_z magnetic field component for the FGM (blue),SCM (red) and the combined data (black). The common frequency used to merge this data was set at 8 Hz.
